# The Committees of Fifteen and Nine

**Published:** 1903-07

**Authors:** 


### THE COMMITTEES OF FIFTEEN AND NINE.

Attention is specially invited, under the proper head, to the very able report of the Committee on Congresses, signed by the Director of Congresses, St. Louis, 1904, Howard J. Rogers, and the Chairman of the Committee on Congresses, Frederick W. Lehmann. It is worthy of careful reading, being a very clear and correct statement of facts relating to the controversy between the Committee of Fifteen appointed by the National Dental Association, at Niagara Falls, August, 1902, to organize the Fourth International Dental Congress, and the Committee of Nine, appointed by the International Dental Federation at Stockholm, Sweden, August, 1902, for the same purpose.

Our readers will find the substance of the conclusions of the committee in the following quotations:

"We are convinced that it is impossible, in view of the present status of the discussion, to make, at the present time, an effective consolidation of the two committees. We are sure that the International Dental Federation would agree with us were they in closer touch with the situation. So large a committee is also impractical from an administrative stand-point. There seems to be nothing left in order to create a successful congress except for the Exposition Company to define the duties of the Committee of Organization of the Exposition, and suggest the line of co-operation of the committee of the International Dental Federation. . . .

"We therefore instruct the Organizing Committee of the Exposition to proceed in the work of organizing the Congress, to obtain the ways and means therefor, to issue invitations, to appoint the necessary committees for preparing the programme, for the entertainment of guests, for nominations, etc., and on such committees to give equal representation in membership to the Committee of Nine, and in all matters to take due care of the rights of every foreign nation and of the obligations due to the International Federation as the authorizing body of the Congress."

This settles a controversy that threatened the success of the proposed congress.

The Committee of Fifteen, at its recent meeting in New York City, accepted, without reserve, the decision of the Committee on Congresses, and will energetically proceed to organize the work upon the lines specified by the aforesaid committee.

The labor of this committee is one that will strain its best efforts. The time between now and the assembling of the Congress in August, 1904, is ample to accomplish satisfactory results, providing the matter is thoroughly systematized. It is to be hoped that the mistakes of the Columbian Congress will not be repeated. There is no room in such a congress for personal ambitions, and these should be frowned upon and forced to occupy a subordinate place.

The writer regards the assertion of the Committee on Congresses, that the International Dental Federation is the "authorizing body of the Congress," to be unwarranted by the facts of history. This body never has had the power to call international dental congresses together. It assumed this under a misapprehension of its duty, and hence naturally followed the much-to-be-regretted controversy between the two committees. It is to be hoped that the St. Louis Congress will arrange, upon a proper basis, future congresses, but this cannot be done by a mere vote. The plan should be submitted to the various representative bodies throughout the world, and upon confirmation by these the matter may be permanently settled and future friction avoided.

